# Surgical Management of Nephrolithiasis in the Bottlenose Dolphin: Collaborations Between the Urologist and Veterinarian

**DOI:** 10.1089/cren.2017.0143

**Published:** 2018-05-01

**Authors:** Roger L. Sur, Jenny M. Meegan, Cynthia R. Smith, Todd Schmitt, James L'Esperance, Dean Hendrikson, Jason R. Woo

**Affiliations:** ^1^ Department of Urology, UC San Diego Health, San Diego, California.; ^2^ National Marine Mammal Foundation, San Diego, California.; ^3^ Seaworld, San Diego, California.; ^4^ Department of Urology, Naval Medical Center, San Diego, California.; ^5^ College of Veterinary Medicine and Biomedical Sciences, Colorado State University, Fort Collins, Colorado.

**Keywords:** nephrolithiasis, urolithiasis, laparoscopy, ureteroscopy, bottlenose dolphin, *Tursiops truncatus*, veterinary medicine

## Abstract

***Background:*** Cohorts of bottlenose (*Tursiops truncatus*) dolphins are at significant risk for nephrolithiasis development. However, effective surgical treatment has been limited due to absence of literature and also familiarity by both veterinarians and urologists. Recently a joint veterinarian and urology team were called to treat local bottlenose dolphins in San Diego, CA, and they performed several cases. The fund of knowledge from these cases is presented for future providers who may be asked to surgically treat these animals.

***Case Presentation:*** Two surgical kidney stone cases were performed by a joint veterinarian and physician team. An effective ureteroscopic stone removal was performed on a 39-year-old female bottlenose dolphin with 9.7 mm distal ureteral calculus. The second case involved laparoscopic ureterolithotomy on a 31-year-old male bottlenose dolphin with a 6-mm right distal ureteral calculus that previously failed retrograde ureteroscopic removal. The stone was not effectively removed laparoscopically as well due to failure to progress associated with operative machinery malfunction. The dolphin was ultimately euthanized.

***Conclusion:*** Despite suboptimal outcome in one case, extremely valuable lessons were learned during both cases. We present our surgical experiences, as well as pertinent anatomical differences, in these animals with the hope that this discussion will facilitate future surgical kidney stone treatment of dolphins.

## Introduction

Bottlenose dolphins, *Tursiops truncatus*, are prone to developing nephrolithiasis.^1^ Veterinarians with specialized equipment and expertise in endourology are not commonly available; therefore, human urologists have been consulted on several cases to jointly perform endourologic procedures in managed dolphins—that is, dolphins not living in the wild. We as urologists expect continued involvement in their care as human urologic techniques are applied to the veterinary domain. There are no prior descriptions of these techniques or of the unique urologic surgical anatomy of *T. truncatus*. The objective of this case report is to present our collective experiences with the hope that others will benefit and improve endourologic care of bottlenose dolphins with nephrolithiasis.

## Presentation of Cases

### Clinical case 1: Ureteroscopy

Flexible ureteroscopy and holmium laser lithotripsy were performed in a 39-year-old female bottlenose dolphin with the U.S. Navy Marine Mammal Program. This animal presented with a unilateral left ureteral partial obstruction caused by a 9.7-mm distal ureteral stone that was detected on abdominal ultrasonography. A 15F digital flexible cystoscope was used with the animal in lateral recumbency under general anesthesia; however, the meatus would not accommodate the scope. A flexible ureteroscope (Karl Storz FLEX-X^C®^ 8.5F digital ureteroscope) was then passed effectively through the meatus/urethra. The bladder demonstrated similar gross bladder mucosa and ureteral orifice location to humans. Through the ureteroscope a 0.035F PTFE-Nitinol guidewire (Boston Scientific Sensor™ wire) was passed into the ureteral orifice, but fluoroscopy was not utilized as dolphin body wall thickness precludes good visualization. The ureteroscope was passed over the wire, and the wire was then removed. Holmium laser lithotripsy was performed with a 200-μm laser fiber until calculi were pulverized into tiny fragments, permitting spontaneous passage. No ureteral stenting was performed due to atraumatic nature of the ureter. The dolphin passed another ureteral calculus several days after surgery and had no other postoperative complications with resolution of hydronephrosis on ultrasound.

### Clinical case 2: Laparoscopy

Herein, we present the first attempted laparoscopic surgery for dolphin stone disease. A 31-year-old male bottlenose dolphin with the U.S. Navy Marine Mammal Program with a history of chronic severe bilateral nephrolithiasis presented with azotemia and cross-sectional imaging consistent with a partially obstructing right distal ureteral stone. A prior, separate attempted flexible retrograde ureteroscopy under general anesthesia was unsuccessful because long tortuous (“loop the loop”) urethral anatomy precluded use of cystoscopes or ureteroscopes. Because of the unique dolphin anatomy and progressive clinical decline despite aggressive medical measures, we opted to perform a laparoscopic ureterolithotomy as a potential life-saving measure.

#### Operative details of laparoscopy-assisted ureterolithotomy

**FIG. 1.**
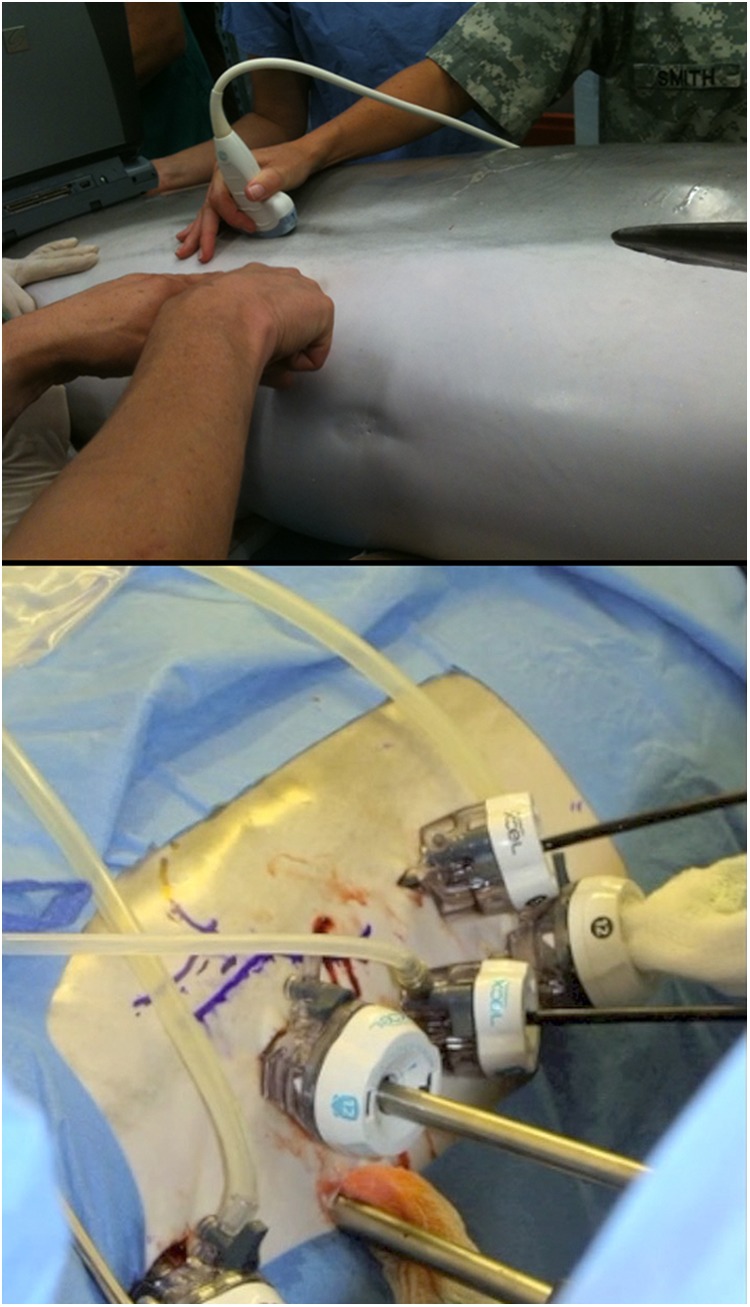
Preoperative trocar planning using ultrasound probe and subsequent trocar placement.

**FIG. 2.**
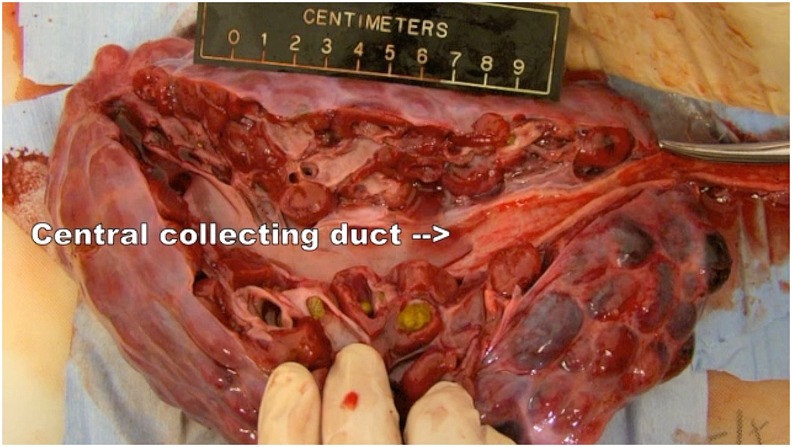
Bivalved kidney showing that dolphin collecting duct is much less capacious than renal pelvis, the human homologous structure.

The dolphin was lifted out of water, beached on mat, and moved to hydraulic table that was padded with closed-cell foam. The dolphin's skin was kept moist with wet towels and fresh water spray. Dolphins were premedicated with a benzodiazepine, and anesthesia was induced with propofol infusion. The dolphin was orotracheally intubated and maintained on gas anesthesia with controlled mechanical ventilation. Transabdominal ultrasound was performed to determine the location of the right kidney, identifying the cranial, caudal, and ventral extent of the kidney to help with portal placement (Fig. 1). A 15-mm skin incision was made at the caudal aspect of the right kidney and ∼10 cm ventral to the ventral aspect of the right kidney. An optical trocar was used to gain peritoneal access and obtain insufflation. The abdominal wall lacked the compliance of the human abdominal wall, making control of pneumoperitoneum challenging. The right kidney was easily identified, and an additional 12-mm port was placed caudally and a 5-mm port cranially. Several other trocar sites were later utilized in an attempt to optimize our laparoscopic approach as our original port placement was not optimal (Fig. 1). Standard laparoscopic instruments were used. The diaphragm, liver, right kidney, right testis, and bowel were easily identified. Other than the intra-abdominal testis which lies more cranial, the surgical landmarks are similar to human anatomy and familiar to a urologist. The peritoneum was incised over the kidney, and the ureter was identified at the caudal aspect of the kidney. A 4-mm longitudinal incision was made into the ureter, and two guidewires were advanced into the ureter and renal pelvis. A flexible ureteroscope was passed into the ureter, and at least four or five intrarenal calices were visualized which were similar in internal appearance to human calices (Fig. 2). Of note during the entire procedure, pneumoperitoneum was intermittently inadequate for visualization due to machinery malfunction, making surgical progress extremely challenging and prolonged. Before being able to evaluate the entire ureter where the ureteral calculus actually was located, the dolphin went into cardiac arrest. Extracorporeal chest compressions were performed, and the patient was resuscitated. Due to the unstable patient condition, the procedure was aborted, and the dolphin was ultimately euthanized due to his poor prognosis. Necropsy demonstrated obstructing ureteral stone just distal to ureterotomy and numerous renal stones.

## Discussion and Literature Review

As with human kidney stone disease, surgical treatment of dolphin kidney stone disease depends on stone size, stone number, location, and patient anatomy. Our experiences highlight the importance of gender as a key component in the decision algorithm. Retrograde endoscopic approach is feasible in female dolphins, which is the ideal surgical treatment approach. However, anatomical differences in male dolphins preclude a retrograde endoscopic approach. Male dolphins pose a significant surgical challenge as the standard approaches for human kidney stone disease have not proven feasible.

**FIG. 3.**
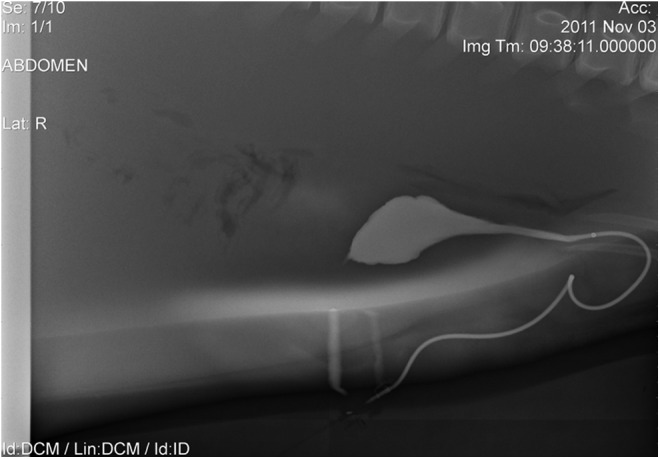
Male bottlenose dolphin urethra with its severe curvature, which complicates retrograde endoscopic approaches. Retrograde cystourethrogram (sagittal view).

Attempts to perform retrograde ureteroscopy have been unsuccessful in male dolphins, due, in part, to the unique male urethral anatomy. The male urethral anatomy poses significant endoscopic challenges due to severe urethral curvature and excessive urethral length of ∼50–70 cm (Fig. 3). While both flexible cystoscope and ureteroscope permitted negotiation of curvature, the long length prevented visualization of the bladder, and the ability to deflect the tip of the scope was lost. Combined laparoscopic endoscopy may likely be the most ideal for male gender. Given the challenges of this combined endoscopic laparoscopic surgical technique, endoscope equipment advances and novel surgical techniques seem warranted to advance the current approaches, such as longer flexible ureteroscopes and methods to handle unique renal anatomy, respectively.

Retrograde ureteroscopy has been effectively performed previously in a female dolphin.^2^ In the study by Schmitt and colleagues,^2^ we described the use of flexible cystoscopic placement of safety and working wires. In the event the meatus does not accommodate a flexible cystoscope, then either a flexible or semirigid ureteroscope can be utilized to place a wire. Semirigid ureteroscopy with holmium:YAG laser lithotripsy of ureteral calculi was effectively performed on the abovementioned female dolphin. The ureter accommodated a 6.9-semirigid ureteroscope (Olympus MRL-6).

Of note, both male and female dolphin urethras appear narrower than the human urethra. Although we were able to access the female urethra using a 12F flexible fiberoptic cystoscope in abovementioned case report, we were unable to pass a 15F digital flexible cystoscope in our female dolphin endoscopic case. Needless to say, the urethral meatus was too narrow for a 21F rigid cystoscope. It is unclear if meatal dilation would permit passage of a rigid cystoscope, but the presence of the caudal peduncle and flukes may obstruct the mobility of a rigid cystoscope.

**FIG. 4.**
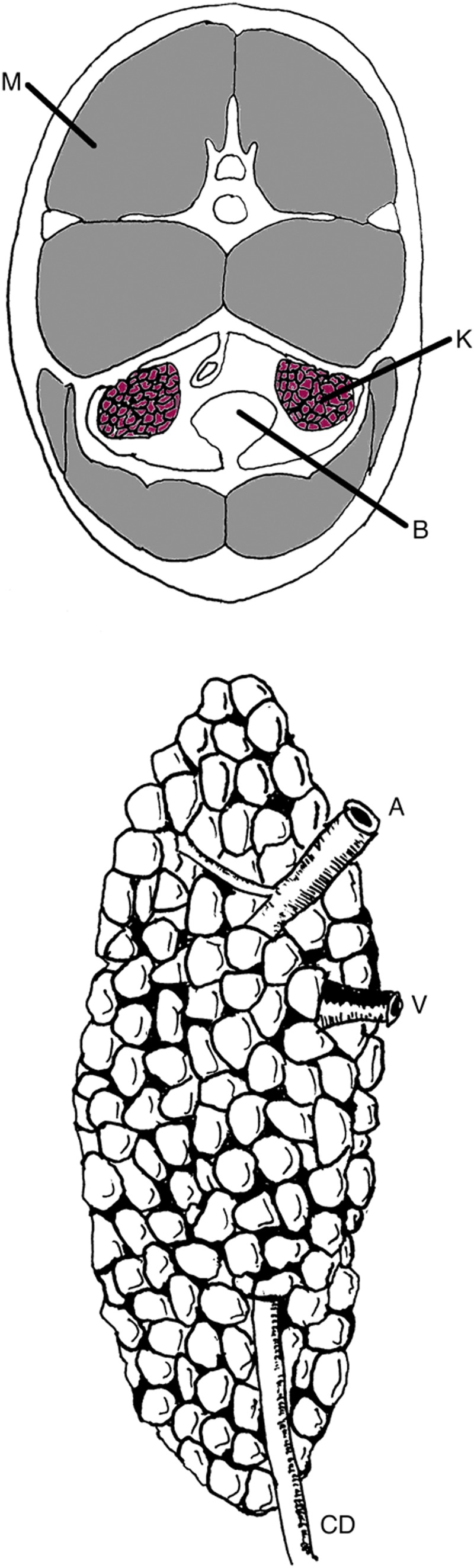
Author's drawing of dolphin cross section. Note the thick axial musculature (M), the ventrally located kidneys (K), and the urinary bladder (B). Dolphin kidney with renicules. Renal vein (V), renal artery (A), and collecting duct (CD).

The most striking difference between human and dolphin kidneys is the renicular structure of dolphin kidneys. These kidneys are made up of hundreds of individual lobes or reniculi (Fig. 4), each of which contains discrete cortical tissue and a single medullary pyramid inserted in a single canaliculus. A central collecting duct is formed by the junction of the excretory canaliculi draining individual reniculi. The collecting duct runs the length of the kidney and exits from the caudal aspect of the kidney as the ureter.^3^

A discussion on ureteral stenting is worth mentioning. During cystoscopic ureteral stent placement in a previously published case report on dolphin treated with semirigid ureteroscopy, the 26 cm stent was undersized for the dolphin ureter and the stent subsequently migrated proximally into the ureter.^2^ Based on these findings, we recommend ureteral stents of at least 28 cm. Table 1 demonstrates known data regarding ureteral lengths.

**Table 1.** Length Measurements of Bottlenose Dolphin Collecting Ducts and Ureters Obtained with Intravenous Contrast CT

	*Range of collecting duct length*	*Range of ureteral length*
Overall (*n* = 5)	11.2–16.7 cm	19.0–25.0 cm
Males (*n* = 4)	11.2–16.7 cm	19.0–23.7 cm
Females (*n* = 1)	11.5–13.0 cm	24.5–25.0 cm

Finally, anesthesia can be extended as long as supportive measures with ventilation, temperature, vascular access, skin hydration, and body support are maintained. Although dolphins are air breathing mammals and as such can breathe out of the water, they do seem to have some issues with lung compliance when not in a water buoyant environment. Therefore, limited time (several hours) out of water is critical.

We unfortunately had no prior surgical literature to guide our approach and this likely contributed to the technical challenge of this case. Nevertheless, we hope that this review will facilitate more efficient and effective future interventions for dolphins with nephrolithiasis. And although there is previous literature providing description of dolphin anatomy, the antiquated nature of the literature merits updating. Moreover, our experience not only provides some novel anatomic findings but also guides future dolphin urologic surgery as it includes clinical correlations to surgical anatomy.

## Conclusion

Despite suboptimal outcome in the latter case, this report demonstrates *extremely* valuable anatomic findings and the feasibility of applying human endoscopic techniques to the Bottlenose dolphin. Given the prevalence of nephrolithiasis among these animals, it is inevitable that these animals will require more joint cooperation among humans. Our collaborations between the urologist and veterinarian will hopefully facilitate more interactions that improve surgical treatment of these animals.
